# Multiscale Material Characterization Based on Single Particle Impact Utilizing Particle-Oriented Peening and Single-Impact Peening

**DOI:** 10.3390/ma13040904

**Published:** 2020-02-18

**Authors:** Nicole Wielki, Matthias Steinbacher, Daniel Meyer

**Affiliations:** 1 Faculty of Production Engineering, University of Bremen and MAPEX Center for Materials and Processes, Badgasteiner Straße 1, 28359 Bremen, Germany; steinbacher@iwt.uni-bremen.de (M.S.); dmeyer@iwt-bremen.de (D.M.); 2 Leibniz Institute for Materials Engineering—IWT, Badgasteiner Straße 3, 28359 Bremen, Germany

**Keywords:** material development, characterization methods, plastic deformation, shot peening, particle-oriented peening, single-impact peening

## Abstract

If conventional methods are used, the development of new structural materials is experience-based, but still intensive in terms of materials, time and cost. As part of the development of a new method for material development and characterization, particle-oriented peening is used in this work. By means of samples of different sizes—but matching microstructures (100Cr6 (AISI 52100), five different material states)—it is examined whether the quantities determined on microscopic samples can be transferred to macroscopic samples. Therefore, peening processes with matching peening parameters but different deformation related aims are compared. While the particle-oriented peening is used to deform the microscopic samples (d = 0.8 mm), the new method of single-impact peening is used to deform the macroscopic samples. For the cross-scale comparison, values characterizing the plastic material deformation (∆l and r_f_, r_c_) are used as well as the particle velocities after the impact influenced by the elasto-plastic material properties. It could be shown that the highly dynamic (material) behavior is comparable in both dimensions. For the future examination of new (unknown) material states it is therefore conceivable to make predictions regarding their material behavior and later on regarding their material properties on the basis of particle-oriented peening of quickly generated microscopic samples e.g., from drop-on-demand processes.

## 1. Introduction

Increasing demands regarding the mechanical properties of highly stressed components used in areas such as aviation requires the development of new, application-oriented materials. At the moment, the development of new structural materials is based on the determination of material properties e.g., tensile, fatigue and hardness measurements. During the phase of material development, the actual suitability of a newly assembled alloy is uncertain, but the tests require large volumes of materials to be synthesized with successive mechanical, thermal or thermomechanical treatment [1,2]. This experience-based but nevertheless iterative approach is therefore time-, material- and cost-intensive. As Zhao showed in his review on combinatorial approaches for material characterization, recent methods for the determination of properties of functional materials are generally carried out on thin films. Therefore, they only allow limited conclusions to be drawn about the behavior of the bulk material [3]. He further explains that diffusion multiples, which are assemblies of several metal blocks arranged in a pre-designed geometry, are suitable for bulk material characterization due to the parallel generation of bulk intermetallic compounds and solid solutions [4]. While in his work published in 2013, he exclusively determines the interdiffusion coefficients and develops a method for simulating these for two-phase systems [5], in previous work, he shows that with the additional performance of nanoindentation tests, knowledge about hardness and the Young’s modulus can also be gained [4,6]. This already allows a first estimation of the material behavior. However, nanoindentation requires a preparation of the specimens and, due to the local testing, allows only limited statements about the properties of larger components.

With the aim of material-reduced material development, investigations can be carried out on specimens of smaller dimensions. Since, unlike thin films, small particles do not neglect the influence of the microstructure and therefore allow conclusions to be drawn concerning their bulk properties [7], a new approach coined “Farbige Zustände“ focuses on the investigation of spherical microscopic particles (diameter ≤ 1.0 mm) [1]. Using, for example, the drop-on-demand technique, where single droplet generation is triggered by introducing a mechanically or pneumatically momentum into the melt, which pushes out the exact droplet volume (cf. [8]), large batches of these samples can be generated comparatively quickly and in a wide range of alloy compositions [9,10,11]. Since standardized procedures such as the mentioned tensile test cannot be carried out using the small specimens, new material property characterizing procedures need to be developed. With the help of these procedures so called descriptors can be determined. They are defined as quantities that describe the material behavior during or as a result of defined processes [12]. Since they are thus directly related to the material properties, the hypothesis is that it is possible to conclude the behavior of components of conventional size based on the investigations performed with the microscopic particles. As a consequence, it should be possible to determine new materials on the basis of investigations performed on the microscopic samples [1].

In conventional shot peening, a large number of particles with a sufficiently high degree of hardness (cf. DIN 8201 [2.2]) and diameters of 0.6 to 2.0 mm [13] are accelerated onto the surface of a workpiece in order to change its surface and subsurface properties [14]. This causes the surface to deform plastically and leads to the formation of residual stresses and a change in hardness, which can have a beneficial effect on properties such as fatigue strength [15,16]. Due to its flexibility, shot peening can be used on complex geometries, making it suitable not only for the finishing of mass-produced components such as springs but also for the finishing of components produced by additive manufacturing. For example, investigations by Sugavaneswaran et al. on direct metal laser sintered stainless steel 316L showed that the plastic deformation of the surface reduced the average surface roughness by 50 % and increased the hardness from 230 HV to 324 HV [17]. AlMangour and Yang were able to show for 17-4 stainless steel that a shot peened sample has improved properties compared to an as-build (by additive manufacturing) sample [18]. Additionally to the plastic deformation of the surface, a very fine microstructure, resulting in more favorable roughness, hardness, compressive yield strength, was obtained [18]. 

The conventional shot peening process, described above, already uses particles of the sizes resulting from the drop on demand process. However, the focus of the process, in which the particles are regarded as non-plastically deforming "tools", is at influencing lager parts of the workpiece and thus changing its properties. By choosing a contact plate of a significantly higher hardness compared to the particles, the focus of the process was shifted by Kämmler et al. [19]. Nevertheless, in this publication still a large number of particles were processed. Based on the investigations of Kämmler et al. a procedure to characterize microscopic samples, the particle-oriented peening, was developed. For each test, a single particle is accelerated to impact on a hardened steel plate (100Cr6 (AISI 52100), approx. 63 HRC) and the velocity before and after the impact as well as the plastic deformation after the test are determined. Already Kämmler et al. were able to show, the plastic deformation of the particles can be correlated to e.g., the hardness of the material, so that differences due to different materials (AlSi12, X210Cr12 (AISI D3)) could be identified [19]. The development of the particle-oriented peening subsequent to Kämmler’s investigations allows an assignment of the quantities determined before, during and after the experiment to a single particle. Furthermore, the acceleration of a single particle prevents particles from colliding in flight, influencing each other, and thus falsifying the result. The experimental setup used within this work for particle-oriented peening was also used to investigate different DSC-treated particles (100Cr6) by Tönjes et al. [20]. Differences due to the varied heat treatment could be detected analyzing e.g., the plastic deformation of the particles. 

Due to the high forming speeds that occur with particle-oriented peening it is of great interest to determine how samples of conventional size (macroscopic samples) behave under comparable conditions, i.e. when a single particle of higher hardness is accelerated to impact on their surface. This variation of the procedure is referred to as single-impact peening in the following and is carried out on the same experimental set-up as the particle-oriented peening which was described above. Although e.g., Clausen has already carried out investigations with individual particles, the focus was on determining the influencing variables in conventional shot peening [21]. Particle velocities, diameters, hardnesses, and angles of impact were varied to examine their influence on the resulting indentation depths. 

**Figure 1:**
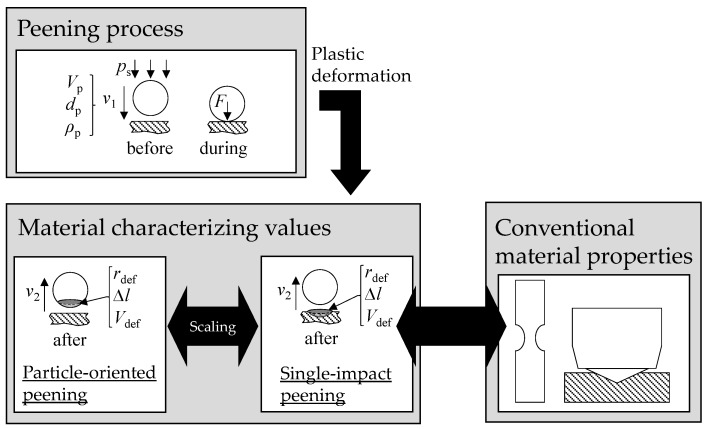
Comparison of the plastic deformation due to a particle-oriented peening process and a conventional shot peening process with a single impacting particle (single-impact peening).

In the current work, single-impact peening is performed to investigate which size effects occur and whether or how scaling of the results of the microscopic and macroscopic samples is possible (cf. Figure 1). Since the dimensions of the latter correspond to those of the samples of conventional material characterization, it should be possible to make statements on the transferability of the knowledge gained from microscopic samples and their correlation with material properties. For cross-scale transferability, it is essential that the values measured on microscopic and macroscopic samples can be related. Therefore, in the current publication, the microstructure of the microscopic and macroscopic samples is adjusted in a similar way by a suitable adaptation of the heat treatment. Currently, the hardness values resulting from the heat treatment are used for the interpretation of the results. In future material development, newly developed alloys can be tested based on microscopic samples as e.g., produced time-efficiently in a drop-on-demand process [8,22]. Thus, the microstructural condition and mechanical material properties of the samples will be unknown in advance. The presented approach aims at revealing the potential of the particle-oriented peening to deduce the material properties, such as hardness on the basis of the values determined for the microscopic samples. Figure 1 shows a scheme of the procedure chosen within this work. Peening processes with matching peening parameters but different deformation related aims are compared. Particles of the same size (d_p_) and density (ρ_p_) are accelerated with the same jet pressure (p_s_). In the case of particle-oriented peening, 100Cr6-particles of different heat treatment (cf. Section 2.2) impact on the hardened steel plate (100Cr6, approx. 63 HRC) and in the case of macroscopic processing (single-impact peening), hardened particles (100Cr6, in average 63 HRC) impact on 100Cr6-plates of different heat treatment (cf. Section 2.2). For both orders of magnitude, the same heat treatments are compared. In each case, the material characterizing values are the deformation resulting from the impact and the velocity of the particle after the impact. The tensile specimen and the hardness test shown schematically (cf. Figure 1 (right side)), representing conventional materials testing, illustrate that, in order to compare the results of particle-oriented peening with conventional material properties it must first be ensured that the findings concerning material behavior are valid across all scales.

## 2. Materials, Methods and Approach

### 2.1. Materials

#### 2.1.1. Particles

**Figure 2:**
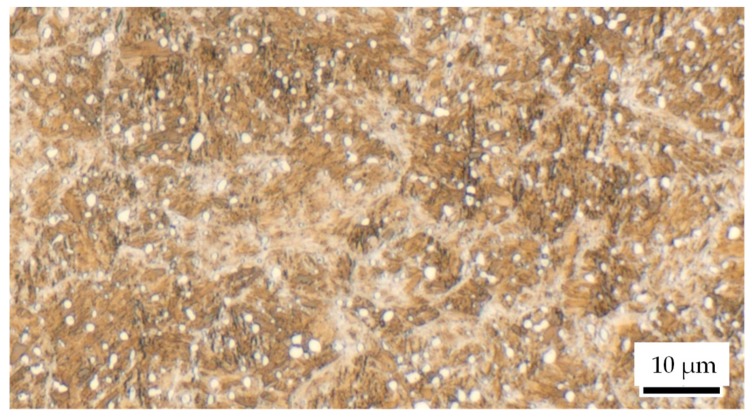
Microstructure of the microscopic samples after the roller ball production process; etched 25–30 s in 3% alcoholic HNO_3_, equatorial cut.

The experiments were carried out with commercially available spherical microscopic samples (diameter d = 0.8 mm) of steel 100Cr6 (AISI 52100 (IHSD-Klarmann, Bamberg, Germany); chemical composition cf. Table 1). In the purchased condition, these samples, generated with a roller ball production process, have a martensitic microstructure with retained austenite and globular carbides (cf. Figure 2). Due to this microstructure, the particles have a hardness of about 60-66 HRC. While this state was used for the single-impact peening, the particles were heat-treated for particle-oriented peening according to Section 2.2.

#### 2.1.2. Impact Plates

**Figure 3:**
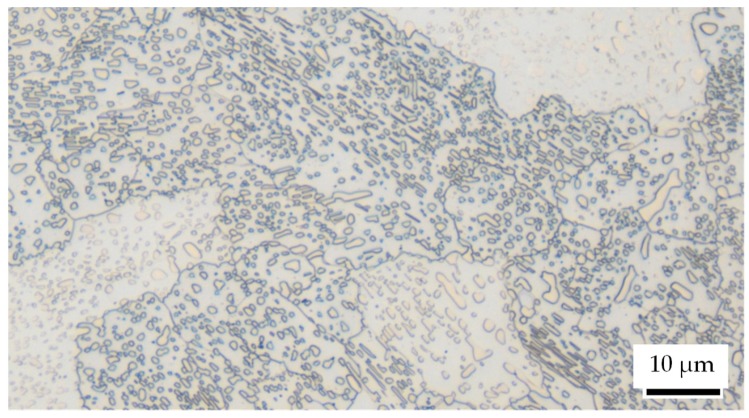
Initial microstructure (before heat treatment) of the macroscopic samples (single-impact peening); etched 25–40 s in 3% alcoholic HNO_3_.

The hardened impact plates for particle-oriented peening as well as the plates used in the single-impact peening were commercially available 100Cr6 (Stahlhandel Gröditz GmbH, Gröditz, Germany). For particle-oriented peening, the hardness of the impact plate (dimensions: 95 mm × 90 mm × 7 mm) was set to approximately 63 HRC to ensure comparability with the martensitic particles. The plates for the single-impact peening (dimensions: 30 mm × 50 mm × 10 mm) were also heat-treated according to Section 2.2. In order to facilitate the analysis of the individual impacts, the surface of the plates was polished before the deformation process. The microstructure supplied is in spherodized state with finely distributed globular cementite within a ferritic matrix (cf. Figure 3). The initial ferrite grain diameter is about 15–30 µm. Without further heat treatment, the material has an average hardness of 189 HV 1 ± 8 HV 1. According to the data provided by the supplier, the chemical composition is listed in Table 2.

### 2.2. Heat Treatment

To vary the material properties, the investigated particles and impact plates were heat-treated in a standard vacuum furnace under varying conditions prior to the deformation processes. Table 3 gives an overview of the parameters used for heat treatment. Due to their larger dimensions and different initial microstructural states, the holding times for the impact plates have been adjusted for each step. In the table, the holding times for the microscopic particles are underlined and the holding times for the impact plates are highlighted in grey. 

**Figure 4:**
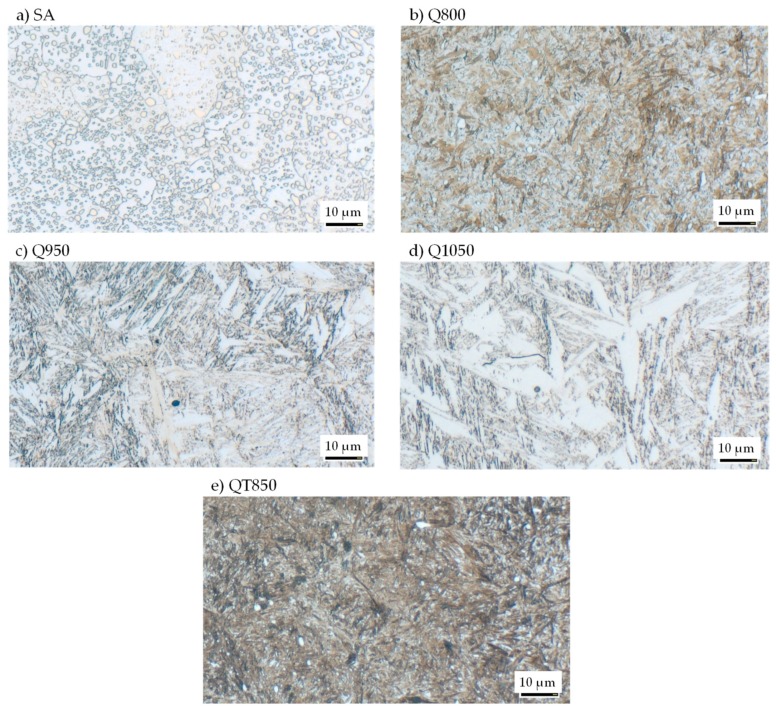
Microstructure of the macroscopic samples for single-impact peening after the heat treatment (**a**) SA, **(b**) Q800, **(c**) Q950, **(d**) Q1050, **(e**) QT); etched 25–60 s in 3% alcoholic HNO_3_.

Figure 4 exemplarily shows the microstructure after heat treatment for the macroscopic workpieces. The annealing treatment (SA) led to a finely distributed spherodized state of cementite within a ferritic matrix. Chromium carbides are also visible. The Q800 (800 °C) heat treatment let to fine martensitic microstructure (martensite needles) with fine carbides. The former austenite grain size can be estimated at about a diameter of 30 µm. Heating the material up to an austenitisation temperature of 950 °C (Q950) led to a dissolution of most of the iron carbides. Figure 4c) is showing a fully martensitic microstructure with a low retained austenite content. The needle martensite is coarser and the retained austenite is light optically visible. Due to the austenitizing in the austenite field above A_cm_ no globular cementite is present any more. The Q1050 (1050 °C) variant is showing a coarse needle martensite microstructure with retained austenite. Because of the high austenitizing temperature, all carbides of iron and chromium carbide type are solute and a pronounced grain grows has occurred. The prior austenite grain size can be estimated to approx. 100 µm diameter. The morphology has changed from fine needle martensite into a coarse needle and stringer martensite with chevron martensite being present in some areas. Figure 4e shows the microstructure of the QT (quenched and tempered) variant. The tempering at 180°C of the quenched martensitic microstructure with very fine dispersed non optical microscopy visible retained austenite was achieved. Additionally, fine dispersed spherical carbides can be seen in the microstructure. Along with the carbides in some instances very little nonmartensitic black etching microstructure constituents can be seen, that are identified as troostite.

### 2.3. Plastic Deformation and Characterisation

**Figure 5:**
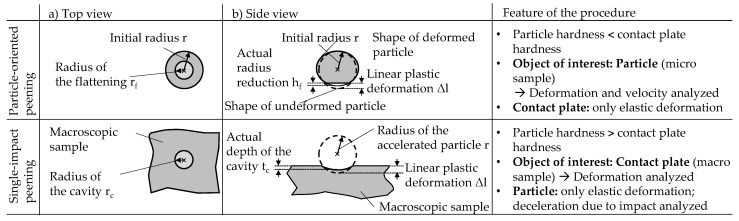
Definition of the determined descriptors (r_f_: radius of the flattening, r_c_: radius of the cavity, ∆l: linear plastic deformation, t_c_: depth of cavity).

A newly established set-up was used for both the particle-oriented and the single-impact peening. It allows to process a large number of particles in a highly defined and reproducible way [19,20,23]. In both cases the particles are accelerated with compressed air (jet pressure p_s_ = 4 bar) to impact on a contact plate which is located at a constant distance of a = 80 mm in front of the nozzle outlet. Depending on the combination of the particle’s and the plate’s material state, the impact causes the former or the latter to deform plastically according to their material properties. At the given peening conditions, the observed plastic deformation allows conclusions to be drawn about the mechanical properties of the samples [19,20]. After the peening process, the plastic deformation is analyzed under a light microscope (Zeiss SteREO.V12, REOObjective.435200-0000-000 objective, Jena, Germany) and the radius of the flattening r_f_ or the radius of the cavity r_c_ is determined as schematically shown in Figure 5. Utilizing the geometric relationship of the sphere segment, the initial radius of the accelerated particle r and the radius of the flattening or the radius of the cavity can be used to calculate the linear plastic deformation Δl. As indicated in Figure 5, it can be assumed that differences occur between the linear plastic deformation and the depth of the cavity t_c_ due to the assumption of an ideal spherical particle and the disregard of the elastic deformation for the single-impact peening. Since this depth cannot be determined by light microscopy, the indentations are additionally analyzed using a laser confocal microscope (Keyence VK-X1000; magnification: 20x, KEYENCE DEUTSCHLAND GmbH, Neu-Isenburg, Germany). Furthermore, for particle-oriented peening, it can be assumed that the use of formula correlations for an ideal sphere segment also leads to deviations between the calculated linear plastic deformation and the actual “radius reduction”. However, since this cannot be determined by aid of a microscope, it must be examined to what extent the knowledge possibly gained on the relationship between ∆l and r_c_ for the single-impact peening can be transferred to the particle-oriented peening. ∆l and r_f_, r_c_ are used for the cross-scale comparison due to their consistent determination.

### 2.4. Particle Velocity

In addition to the use of the determined plastic deformation, the particle velocity after the impact v_2_ can be used for a cross-scale comparison. This velocity as well as the reduction of the particle velocity ∆v_p_ depends on the material pairing, i.e., the properties of particle and plate at the moment of the impact. If a similar amount of kinetic energy is transferred to the plastic deformation of one of the two components, a comparable deceleration of the particles can be assumed. To ensure this, the velocity of the particles before the impact v_1_ is also determined. The velocities are measured utilizing a system consisting of a light barrier (manufactured by “FOS Messtechnik GmbH”, Schacht-Audorf, Germany), two stroboscopes (“HELIO-STROB micro2” manufactured by “ELMED Messtechnik GmbH”, exposure frequency: 4000 Hz) and a monochrome camera (type “DMK 5 37BUX250” manufactured by “The Imaging Source Europe GmbH”, Bremen, Germany).

## 3. Results

**Figure 6:**
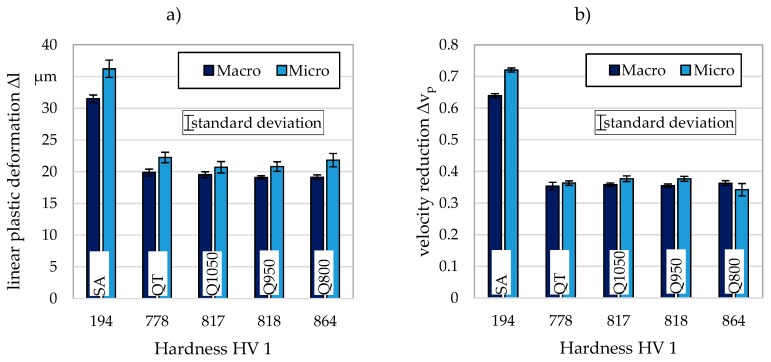
Comparison of (**a**) the linear plastic deformation and (**b**) the velocity reduction due to the impact; Macro: Macroscopic value (single-impact peening; n ≥ 5), Micro: Microscopic value (particle-oriented peening; n = 25).

During particle-oriented and single-impact peening, comparable particle velocities could be achieved before impact. For particle-oriented peening an average particle velocity of 55.73 m/s ± 0.97 m/s (number of tests carried out: n = 45) was determined at a jet pressure of 4 bar and for single-impact peening a particle velocity of 56.72 m/s + 1.16 (n = 35) was measured. As explained in Section 2, this allows plastic deformations and velocity reductions to be compared across scales. Figure 6 compares the linear plastic deformation (a) and the velocity reduction (b) determined for the single-impact (Macro) and the particle-oriented (Micro) peening in dependence of the hardness HV 1 determined on the macroscopic samples for each material state. Considering the linear plastic deformation, the highest values for both microscopic and macroscopic samples were determined for the soft annealed condition. This observation corresponds to the results of Steinbacher et al., who determined various descriptors on microscopic particles [12]. 

With increasing hardness from the soft annealed to the hardened states, the plastic deformation decreases. While this decrease is visible with only a small deviation for the linear plastic deformation determined for the macroscopic samples, the values determined for the harder material states of the microscopic samples first decrease before a higher plastic deformation is visible again for the hardest material state Q800. Due to the higher standard deviation determined for the microscopic samples, a trend can only be recognized to a limited extent. This can be traced back to the hardness of the hardened as well as of the quenched and tempered material conditions being of the same order of magnitude. In the range of high hardness, probably also due to the high velocity of the impact, the resistance to plastic deformation is so high that a differentiation of the materials states utilizing the quantities determined with both peening variants is only possible to a limited extent. For all investigated material states, higher values of the linear plastic deformation were obtained for the microscopic samples. For Q1050 the value is 6%, for Q950 8%, for QT 11%, for Q800 12% and for SA 13% higher than the value determined for the macroscopic sample. 

Considering the velocity reduction, the value determined for SA again shows a clear difference to the values determined for the other material states (cf. [12]). Here, too, the value determined for the microscopic samples usually exceeds the value determined for the macroscopic samples. The highest deviation between the two orders of magnitude is 11% for the soft annealed state. For QT the value determined for the microscopic samples is 3%, for Q1050 5% and for Q950 6% higher than the value determined for the macroscopic samples. Only for the hardest condition (Q800), the value obtained for the microscopic sample is 6% below the value determined for the macroscopic sample. Regarding the microscopic samples, the velocity reduction determined for this material condition shows the highest standard deviation.

**Figure 7:**
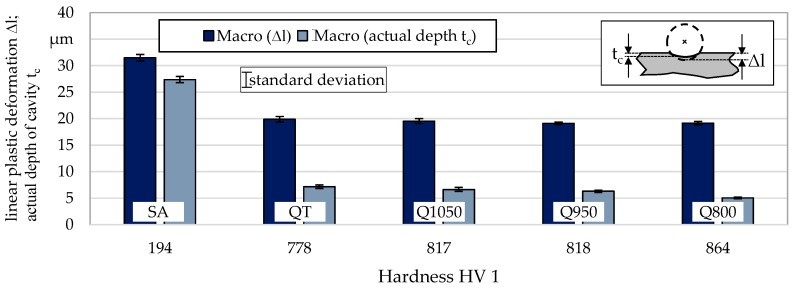
Comparison of the linear plastic deformation and the actual depth of the cavity determined for the macroscopic samples (single-impact peening; n ≥ 5).

There is a difference between the linear plastic deformation ∆l and the actual depth of the cavity t_c_ determined for the macroscopic samples. This difference can be traced back to the idealized assumption of a sphere segment for the calculation of linear plastic deformation (cf. Section 2.3), which does not take into account the elasto-plastic deformation of the whole peening particle and the elastic reshaping of the sample material. While the former is determined based on the measured indentation diameter, the latter is measured by a laser confocal microscope (cf. Section 2.3). Figure 7 compares both values for all material states. It can be seen that in all cases the actual depth of the cavity is less than the value calculated theoretically (based on the deformation radius). While this difference is only 13% for the annealed (SA) condition, the linear plastic deformations for the other conditions is 64% (QT), 66% (Q1050), 67% (Q950), and 74% (Q800) higher than the actual depth. The deviation of the calculation from the actual depth of the cavity thus increases with increasing material hardness. The hard material reveals a more pronounced elastic behavior. This can be explained by the fact that with increasing hardness not only the total deformation ability of the material is reduced, but also the plastic amount of the total deformation ability. Furthermore, the lower measured depth may be related to the elastic deformation of the peened particle at the moment of impact which is not taken into account for the calculations.

## 4. Discussion

Regarding the plastic deformation as a result of the impact, a consistent determination of the linear plastic deformation shows that for both orders of magnitude, with maximum deviations of 13%, comparable results are obtained. The determined deformation decreases with increasing hardness. Due to comparable hardness values for the hardened as well as the quenched and tempered condition and the increased standard deviations of the values determined for the microscopic samples, this trend can only be clearly seen for the linear plastic deformation of the macroscopic samples. The higher values of the plastic deformation determined for the particle-oriented peening may be explained by the different stress distributions in the samples (microscopic ≠ macroscopic) in the moment of contact and the fact that in the case of single-impact peening, a higher resistance to the deformation due to the surrounding material occurs. This surrounding material could also lead to another elastic reshaping of the specimen, which is why the investigation of the actual cavity depth can only be an indication for the particle-oriented peening. The observation that samples of the same material, the same heat treatment, and grain size, but of different dimensions, may behave differently can be attributed to the size effects reported by e.g., Vollertsen et al. [24]. According to Vollertsen, the yield stress of the material, among other variables, is influenced by various size effects. Furthermore, the size effects in forming influence the accuracy of the parts [24], including spring-back (cf. [25]). This supports the hypothesis of a different elastic deformation behavior during single-impact peening compared to particle-oriented peening.

For the single-impact peening, it was shown that with increasing material hardness there are larger differences between the theoretically calculated linear plastic deformation and the actual depth of the cavity. The theoretical values were between 13% and 74% higher than the measured depths. This means that in the case of a predominantly plastic deformation, reality is better represented by the calculation than in the case of a mostly elastic deformation. Together with the assumption that, not only the total deformation ability but also its plastic amount decreases with increasing hardness, this suggests that the linear plastic deformation also exceeds the actual radius reduction in the case of the particle-oriented peening of hard material states. This is supported by the work of Sawa, who was able to show that the plastic deformation work decreases with increasing hardness for various materials, such as copper or carbon tool steel [26]. For the soft material state, plastic deformation seems to occur easier in the area of the flattening, since in contrast to a hard particle, the elastic deformation of the entire particle is less pronounced. 

**Figure 8:**
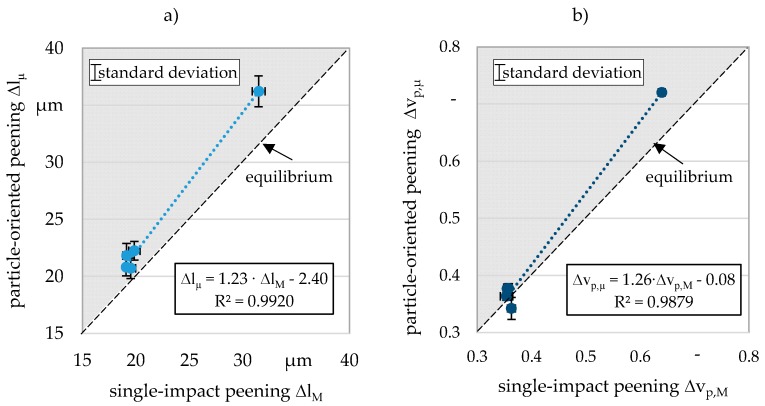
Determination of the functional scaling relationships (**a**) linear plastic deformation, (**b**) velocity reduction.

Considering the velocity reduction, it was possible to determine comparable values for both the particle-oriented peening and single-impact peening. The maximum deviation of 11% determined for the soft-annealed condition indicates that the macroscopic material is elastically deformed differently than the microscopic particle. Figure 8 shows the results determined for the microscopic and the macroscopic samples plotted against each other. The functional correlations that can be determined with a high coefficient of determination make it possible to predict the behavior of the macroscopic samples on the basis of microscopic experiments. For both the linear plastic deformation and velocity reduction, functions with a slope of about 1.2 result (cf. Figure 8). As can be seen by the line of the equilibrium, the plotted points are located in the upper left part of the diagram (cf. grey background). For any value determined utilizing the single-impact peening, a slightly higher value results for particle-oriented peening. The differences also seem to increase with increasing absolute values. This observation, already made during the discussion of Figure 6, leads to the conclusion that the material surrounding the cavity leads to a less pronounced plastic deformation in the case of the macroscopic samples and thus also to a lower deceleration of the particles as a result of the impact. The deformation of the microscopic particles thus faces a lower resistance, which could alternatively also be attributed to the stresses differing from the macroscopic samples.

## 5. Conclusions and Outlook

In this publication, five different material states (SA–soft annealed; Q800, Q 950, Q1050–hardened; QT–quenched and tempered) of the material 100Cr6 were investigated at two different scales. For this purpose, particle-oriented peening (microscopic) and single-impact peening (macroscopic) were used. Regarding the former, the impact plate was kept constant (hardened 100Cr6–66 HRC) and particles (d = 0.8 mm) of the five described states were used. For the latter, the particles were kept constant (hardened 100Cr6–66 HRC) and impact plates of the five material states were investigated. The aim of this work was to investigate whether or how a scaling of the results is possible, utilizing the descriptors determined analyzing microscopic and macroscopic samples. Thereby the validity of transferring the knowledge gained from microscopic samples to material properties was analyzed. On basis of the determined plastic deformation and the velocity reductions due to the impact the major findings of this work are:

- The highly dynamic (material) behavior is comparable in both dimensions.
- The linear plastic deformation is suitable for a cross-scale comparison, although the actual depth of the resulting cavity (single-impact peening) is increasingly overestimated by the theoretically calculated linear plastic deformation with rising material hardness.
- A prediction of the material behavior of components with larger dimensions based on the particle-oriented peening of microscopic specimens is possible based on the identified functional relationships between the descriptive values determined for both peening processes.
- In the future, this could possibly be used to examine new (unknown) material states without the need to analyze the microstructure or to measure the material hardness. Thus, conclusions about the highly dynamic material behavior might be possible, saving material, time, and costs.

By investigating further material states that differ more clearly in hardness, it can be determined whether the above-mentioned overestimation follows a functional relationship. The transferability of such a relationship to the particle-oriented peening could then be examined, by e.g., a three-dimensional scan of the deformed particles. Furthermore, the investigation of additional states could be used to validate the scaling functions found. Whether these scaling functions are influenced by the alloy composition needs to be examined by investigations of other materials.

In this publication, it was shown that a scaling from particle-oriented to single-impact peening is possible. In future work, the transferability of the quantities determined for the macroscopic samples to further conventional material properties must be investigated.

## Figures and Tables

**Table 1:** Average chemical composition of the roller balls used for the experimental work cf. [12].

		Chemical Composition in wt.%							
Material		Fe	C	Cr	Mn	Ni	P	S	Si
AISI 52100 (cf. [12])		bal.	1.07	1.31	0.35	0.17	--	0.0018	0.35
DIN EN ISO 683-17:2000-04	min max	bal.	0.93 1.05	1.35 1.60	0.25 0.45	0.00 0.40	- 0.025	- 0.015	0.15 0.35

**Table 2:** Chemical composition of the plates for singe-impact peening.

	Chemical Composition in wt.%							
Material	Fe	C	Cr	Mn	Ni	P	S	Si
AISI 52100	bal.	0.98	1.43	0.31	0.14	0.016	0.009	0.26

**Table 3:** Heat treatments (micro: microscopic particles, macro: macroscopic samples).

	Hardening Parameter	Quenching Parameter	Tempering Parameter
**SA-micro**	600 °C (30 min), 800 °C (3 h), 690 °C (3 h)	Furnace cooling	-
**SA-macro**	600 °C (30 min), 800 °C (5 h), 690 °C (5 h)	Furnace cooling	
**Q800-micro**	800 °C (1 h)	N_2_ 8 bar	-
**Q800-macro**	800 °C (2–3 h)	Oil 60 °C agitated	
**Q950-micro**	950 °C (1 h)	N_2_ 8 bar	-
**Q950-macro**	950 °C (2–3 h)	Oil 60 °C agitated	
**Q1050-micro**	1050 °C (1 h)	N_2_ 8 bar	-
**Q1050-macro**	1050 °C (2–3 h)	Oil 60 °C agitated	-
**QT-micro**	850 °C (1 h)	N_2_ 8 bar	180 °C (2 h) in atmosphere,
**QT-macro**	850° C (2–3 h)	Water RT	180 °C (2–3 h) in atmosphere
